# Enhancing Cellular Infiltration on Fluffy Polyaniline-Based Electrospun Nanofibers

**DOI:** 10.3389/fbioe.2021.641371

**Published:** 2021-06-09

**Authors:** Zohreh Daraeinejad, Iman Shabani

**Affiliations:** Department of Biomedical Engineering, Amirkabir University of Technology (Tehran Polytechnic), Tehran, Iran

**Keywords:** polyaniline nanofibers, electrospinning, cellular infiltration, tissue engineering, solvent, three-dimensional scaffold

## Abstract

Despite the unique properties of polyaniline (PANI), the processability of this smart polymer is associated with challenges. Particularly, it is very difficult to prepare PANI nanofibers due to poor solubility, high charge density, and rigid backbone. The most common approach for solving this problem is blending PANI with a carrier polymer. Furthermore, the major limitations of nanofibers for tissue engineering applications are their low porosity and two-dimensional (2D) structure. In this study, conductive nanofibers were fabricated through electrospinning of PANI/poly(ether sulfone) (PES) with different solvents including dimethyl sulfoxide (DMSO), N-methyl-2-pyrrolidone (NMP), and hexafluoroisopropanol (HFIP). The effect of solvent, carrier polymer (PES), and PANI content on formation of 3D conductive nanofibers with appropriate porosity were investigated. It was shown that a solvent with suitable properties should be selected in such a way that the composite nanofibers can be electrospun at the lowest concentration of PES. In this way, the ratio of PANI increased in the scaffold, the electrical conductivity of nanofibers enhanced, and the flat 2D structure of scaffold changed to a fluffy 3D structure. Among the three studied solvents, HFIP with the lowest boiling point and the lowest surface tension was the best solvent for the fabrication of PANI/PES nanofibers. PES could be electrospun at a concentration of 9% w/w in HFIP, while the optimum percentage of PES in DMSO and NMP was above 23% w/w to produce uniform nanofibers. 3D nanofibrous scaffold obtained from 0.5% PANI/9% PES/HFIP solution with electrical conductivity of 3.7 × 10^–5^ S/Cm and porosity of 92.81 ± 1.23%. Cell infiltration into the 3D nanofibers with low packing density improved compared to densely packed 2D nanofibers.

## Graphical Abstract

Electrospun PES/PANI nanofibers were fabricated with three solvents and different PES concentrations. HFIP with the lowest boiling point and surface tension was the best solvent for preparing highly conductive fluffy PANI nanofibers in comparison to the other solvents. 3D conductive nanofibers supported cellular infiltration and proliferation.

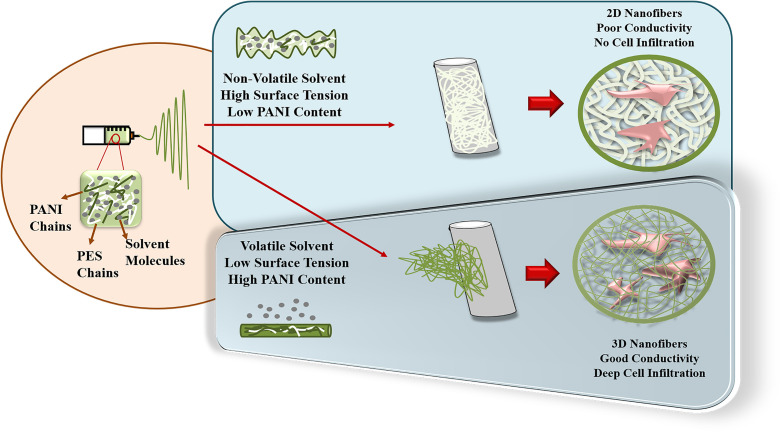

## Introduction

The role of conductive polymers in tissue engineering became more prominent with the fabrication of conductive polymeric nanofibers due to their unique properties and their similarity to the native extracellular matrix (ECM) morphology (Ashtari et al., 2019). An ideal scaffold should mimic the chemical, mechanical, electrical, and topographical function of ECM as much as possible. One of the most important properties of ECM is its nanoscale morphology which affects almost all cellular behaviors (Seyedjafari et al., 2010). Furthermore, it is possible to provide a semiconductive environment for the cells with introducing conductive polymers to the tissue engineering field which transmits electrical signals to electroactive cells such as nerve, muscle, heart, and bone (Guo and Ma, 2018).

Among conductive polymers, PANI has attracted the attention of many researchers due to its facile preparation, low cost, high electrical conductivity, low cytotoxicity, and environmental stability (Qazi et al., 2014; Zare et al., 2019). There are three ways to prepare conductive PANI nanofibers as template-free synthesis, deposition of PANI on template nanofibers, and spinning. Among them, electrospinning is currently the most promising and versatile technique to develop PANI-based nanofibers for tissue engineering scaffolds (Lee, 2013).

The size, microstructure, and general properties of electrospun nanofibers are controlled by different parameters. These are divided into three general groups of parameters including solution, processing, and environmental parameters. Solution parameter is the one play the most important role in the processability of the polymers (Pillay et al., 2013; Angel et al., 2020). It is difficult to prepare a suitable solution and to produce uniform PANI nanofibers due to poor solubility, high charge density, and rigidity of the PANI backbone. So, the electrospinning of PANI is accompanied by many challenges. Various approaches have been reported to defeat this problem (Lee, 2013; Liao et al., 2019). The most common approach is blending PANI with a more flexible, high molecular weight polymer that serves as a processing aid (Skotheim and Reynolds, 2006). Blending non-conducting polymers with PANI is one of the most effective ways to fabricate PANI-based nanofibers. However, the resulting nanofibers have much lower conductivity than neat PANI due to dilution of the conducting component (Zhang and Rutledge, 2012). Therefore, the amount of carrier polymer in PANI-based nanofibers is critical.

The pore size of electrospun nanofibers is less than 10 μm so that the cells cannot easily infiltrate the nanofibers and form a 3D structure such as natural ECM. The limitation of traditional electrospinning is that the nanofibers are deposited tightly on the collector, resulting in a low-porosity sheet-like scaffold. Porosity is a key property in tissue-engineered scaffolds. The porosity and pore size of the scaffold affect the cellular behaviors such as cell spreading, migration, and proliferation. They also affect the exchange of nutrients and waste between the scaffold and the surrounding environment. Numerous strategies have been introduced to overcome this challenge including tuning electrospinning parameters, using sacrificial components, manipulating collector structure, wet electrospinning, ultrasonication, and inclusion of biological factors (Khorshidi et al., 2016). Changing the biochemical and structural properties of nanofibers in our previous works improved the cell infiltration to nanofibrous scaffolds. In the first approach, collagen as a bioactive molecule was used to attract cell infiltration into collagen-grafted PES nanofibers (Shabani et al., 2009, 2011). In another study, the issue of fiber packing density was addressed to fabricate 3D electrospun nanofibers through a simple modification of the electrospinning process using an array of focused lightbulbs. Localized heat generated by a series of halogen lamps in the path of the jet close to the collector, increased the evaporation rate of the solvent (Shabani et al., 2012). Furthermore, the two approach were combined to prepare a 3D collagen-grafted PES nanofibrous scaffold. The scaffold showed the potential for feeder-free culture of pluripotent stem cells because of its 3D structure and bioactivity which enhanced pluripotency, proliferation, differentiation, and infiltration of embryonic stem cells (Hashemi et al., 2011).

In this study, the role of solvent in the preparation of highly conductive 3D PANI nanofibers is investigated. The choice of solvent is very important to prepare an electrospinning solution consist of a combination of PANI and a carrier polymer. Three important considerations to selecting an appropriate solvent are the solubility of the polymers in the solvent, the effect of solvent on the conductivity of PANI, and the properties of the solvent such as boiling point, dielectric constant, and surface tension which affect the electrospinnability of the solution. A suitable solvent for PANI is a solvent that can dissolve the highest percentage of PANI and can change the molecular conformation of the PANI from compact coil to straight chain (MacDiarmid and Epstein, 1995; Zhang and Rutledge, 2012). In other words, the solvent should act as a secondary dopant and help to increase PANI conductivity. Solvents that act as second dopants for PANI include m-cresol, p-cresol, 2-chlorophenol, 3-ethylphenol, 2-fluorophenol, and HFIP (Pud et al., 2003; Yonehara and Goto, 2020). The solvent should also be selected in such a way that electrospinning is possible with a minimum carrier polymer concentration. This is crucial for PANI-based composite nanofibers because increasing the amount of carrier polymer leads to the decrease in the conductivity of the final nanofibers (Song et al., 2018).

Generally, solvent and carrier polymer are two key factors influencing the preparation of electrospun PANI-based nanofibers. Herein, the effect of DMSO, NMP, and HFIP as the solvents on electrospinnability, morphology, and conductivity of PES/PANI nanofibers were investigated. It was also represented that a suitable solvent may lead to a 3D fluffy structure with a good cell penetration. To the best of our knowledge, this is the first report investigating the effect of solvent on the formation of 3D PANI/PES nanofibers with enhanced cell infiltration.

## Materials and Methods

### Nanofibers Fabrication

Electrospinning was used to prepare nanofibers according to a previously reported method (Mohammadi Amirabad et al., 2017). For the fabrication of PES/PANI nanofibers, 5 mg/mL PANI-emeraldine base (PANI-EB; average Mw∼5,000; Sigma-Aldrich) and 5 mg/mL Camphor-10-sulfonic acid (β) (CSA; Sigma-Aldrich) was separately dissolved in dimethyl sulfoxide (DMSO; Merck), N-methyl-2-pyrrolidone (NMP; Merck), and hexafluoroisopropanol (HFIP; Sigma-Aldrich) for 24 h at room temperature. Then PES (average Mw∼58,000; Ultrason^®^, Germany) was dissolved at concentrations of 9, 15, and 23% w/w in the PANI solutions for 24 h. A 21G blunt needle was selected, the constant flow rate was set to 0.5 mL/h, the distance between the needle and the steel grounded collector was 16 Cm, and the voltage was kept at 25 kV. All the electrospinning parameters were the same for the three conductive samples except the solvent type.

### Nanofibers Characterization

#### Morphology

Morphology of the PES/PANI-CPSA nanofibers (PPC) with different solvents and carrier polymer concentration was determined using scanning electron microscopy (SEM; AIS2100, Seron Technology) at an accelerating voltage of 20 kV after sputter coating with Au particles. The average diameter of the nanofibers was calculated from SEM images using image processing software (ImageJ) from at least 70 randomly selected fibers and expressed as mean ± SD.

#### Conductivity

The two-point probe method was used for measuring the electrical conductivity of the conductive samples (2601A, Keithley Instruments) (Koysuren, 2012).

#### Porosity

Scaffold porosity was measured via three different methods named gravimetry, liquid intrusion, and image processing. For gravimetry, five circular samples with 15 mm diameter were used. The estimated porosity of each sample was calculated by the following equation:

P(%)=1-(calculatedmembranedensity/knownmaterialdensity)×100.

For liquid intrusion, the circular dry samples were weighed, sub-merged in absolute ethanol as an intruding liquid, left overnight on a shaker incubator to allow ethanol flow into the void spaces, wiped with tissue paper, and weighed again immediately. Thereafter, the porosity was calculated by the following equation:

P%=V/EtOH(V+EtOHV)s×100

where V_*EtOH*_ is the ratio between mass change after liquid intrusion and ethanol density and Vs is scaffold mass to density ratio. For image processing, porosity and average pore area of electrospun scaffolds were quantified from SEM micrographs using image processing software (ImageJ).

### Cell Culture Study

3T3 fibroblast cells were cultured within T75 flasks with Dulbecco’s modified Eagle’s medium (DMEM) supplemented with 10% fetal bovine serum and 1% antibiotic (penicillin/streptomycin) at 37°C with 5% humidified CO_2_.

#### Cell Morphology on the Scaffolds

The nanofibrous mats were cut into 1.5 cm diameter circular scaffolds to be placed in 24-well tissue culture polystyrene (TCP) and sterilized in 70% ethanol. An initial density of 4 × 10^4^ cells per well was suspended in 400 μL of medium and seeded onto the scaffolds. Cell adhesion and infiltration into the nanofibrous scaffolds were assessed by screening cell morphology on the scaffolds by SEM. Cell-seeded scaffolds were rinsed twice with phosphate-buffered saline (1x PBS) after 48 h to remove non-adherent cells, fixed with 2.5% glutaraldehyde for 1 h, rinsed again with PBS, dehydrated in gradient concentrations of ethanol, and finally left to dry at room temperature. The dried samples were sputter-coated with gold, and observed under SEM.

#### MTT Assay

The proliferation of 3T3 cells on nanofibrous scaffolds was evaluated via MTT assay. The sterilized scaffolds were placed in a 24-well culture plate. The cells were seeded on to the mats and control (TCP) with a density of 10^4^ cells per well and incubated at 37°C and 5% CO_2_. After 1, 3, and 7 days of cell seeding, 50 μL of MTT solution (5 mg/mL in DMEM) was added to each well followed by incubation at 37°C for 3.5 h. For dissolution of the intracellular formazan, the supernatant was removed and isopropanol (Merck) was added. The absorbance at 570 nm was read spectrophotometrically.

### Statistical Analysis

All experiments were conducted at least three times. Data are expressed as mean ± standard deviation (SD). One-way analysis of variance (ANOVA) was used to compare the results. The *P*-value for statistical significance is defined as *p* < 0.05. All statistical analysis was performed using SPSS software.

## Results

### Morphology of the Nanofibers

**FIGURE 1:**
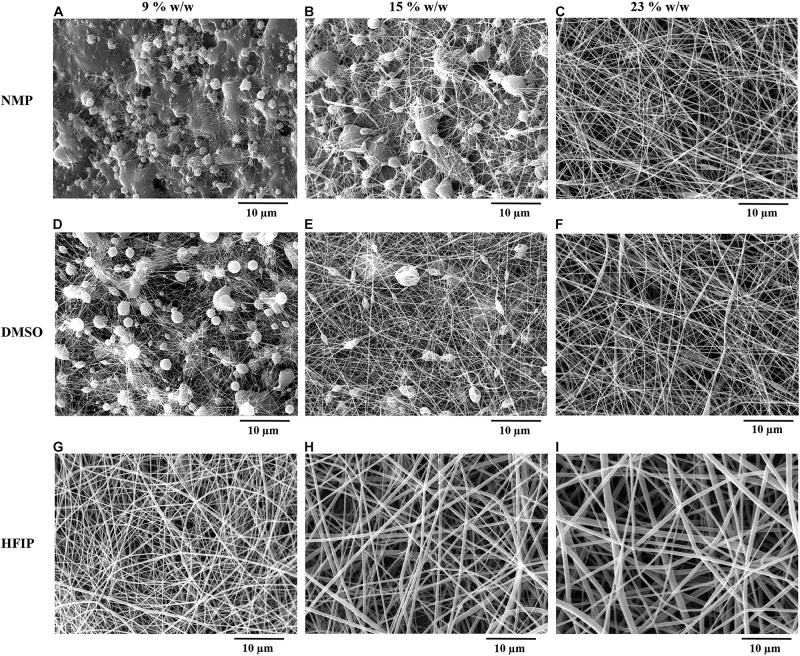
Morphology of electrospun PPC nanofibers prepared with NMP **(A–C)**, DMSO **(D–F)**, and HFIP **(G–I)** in different concentrations of PES [9% w/w **(A,D,G)**, 15% w/w **(B,E,H)**, 23% w/w **(C,F,I)**], (PANI concentration: 0.5% w/w).

The SEM images of electrospun PPC nanofibers prepared with DMSO, NMP, and HFIP at different PES concentrations (9, 15, and 23% w/w) are shown in Figure 1. It is obvious that at the low concentration of PES in DMSO and NMP (9 and 15% w/w), electrospinning and electrospraying occurred simultaneously and there were many particles in these scaffolds (Figures 1A,B,D,E). Fabrication of particle-free nanofibers started at 23% w/w PES concentration, yet nanofibers were not completely uniform and a few beads existed in the nanofibers (Figures 1C,F). On the other hand, electrospinning of PPC solutions in HFIP was successful at all PES concentrations (Figures 1G–I).

**FIGURE 2:**
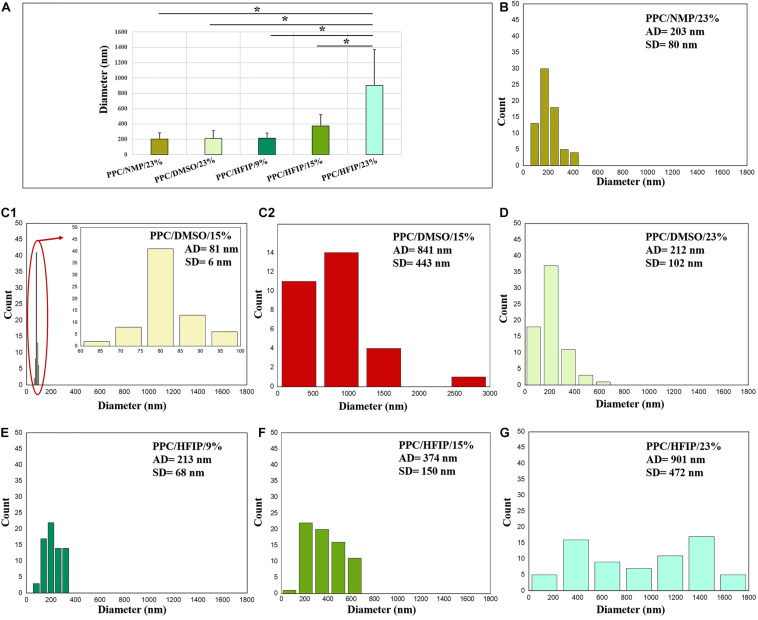
Diameter distribution of electrospun PPC nanofibers: **(A)** Comparison of five nanofibrous sample, **(B)** PPC/NMP/23%, **(C_1_)** PPC/DMSO/15% (nanofibers), **(C_2_)** PPC/DMSO/15% (beads), **(D)** PPC/DMSO/23%, **(E)** PPC/HFIP/9%, **(F)** PPC/HFIP/15%, **(G)** PPC/HFIP/23%. (*Significant differences between PPC/HFIP/23% and other nanofibers diameter, *P* < 0.05). The diagram inside the red ellipse is shown in magnified form.

The diameter distributions of electrospun nanofibers are also shown in Figure 2. The diameter of PPC/HFIP nanofibers decreased by decreasing the amount of PES in the solution, changing from 901 ± 472 nm to 213 ± 68 nm. Furthermore, the diameter of nanofibers prepared with DMSO and NMP at 23% w/w PES concentration was approximately equal to the diameter of nanofibers prepared with HFIP at PES concentration of 9% w/w. Similarly, the larger fibers obtained with HFIP in 23% w/w PES concentration compared to the other two solvents. Considering the nanofiber diameter distribution in Figure 2, high SD from the mean diameter was observed in PPC/DMSO/23%, PPC/NMP/23%, PPC/HFIP/15%, and PPC/HFIP/23% samples. Interestingly, PPC/HFIP/23% fibers showed a bimodal distribution of fibers’ diameter. Two populations of fibers were produced by increasing the solution concentration to 23% w/w. Smaller nanofibers’ diameter is approximately one-third of the larger nanofibers’ diameter.

**FIGURE 3:**
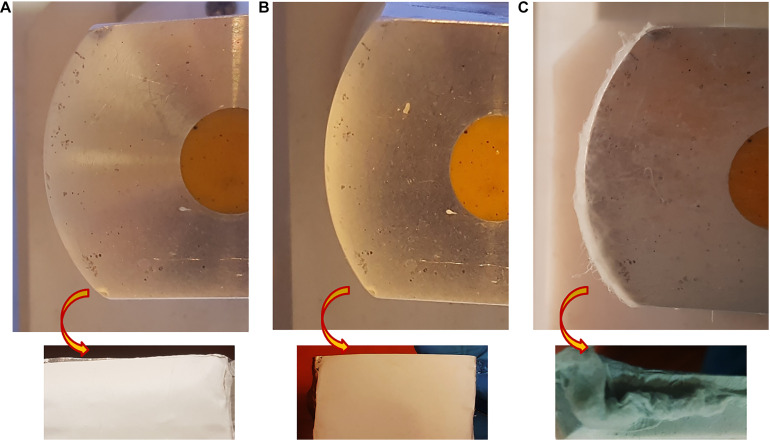
Digital photograph of nanofibers deposited on the collector. **(A)** PPC/NMP/23%, **(B)** PPC/DMSO/23%, **(C)** PPC/HFIP/9% (After 2 h of electrospinning). The images above are of nanofibers deposited on the collectors from the side view. The images below are of nanofibers isolated from the collectors from the top view.

The macroscopic image of 3D and 2D nanofibers are presented in Figure 3. In the PPC/HFIP/9% sample, the fluffy nanofibers were located on top of each other to form a 3D nanofibrous structure while nanofibers were completely compacted on top of each other and formed a thin nanofibrous layer in the PPC/DMSO/23% and PPC/NMP/23% samples.

### Conductivity of the Nanofibers

The conductivity of the nanofibers is shown in Table 1. The higher the percentage of PES in the nanofibers, the lower the conductivity. It was observed that the conductivity of nanofibers prepared with HFIP was higher than those prepared with DMSO and NMP. Furthermore, the conductivity of nanofibers prepared with NMP is higher than PPC/DMSO samples. Only samples containing 9% w/w PES had acceptable conductivity among the prepared nanofibers. However, the morphology of PPC/DMSO/9% and PPC/NMP/9% were not suitable. The three highlighted samples in Table 1 were used for cellular studies.

**TABLE 1:** Conductivity of electrospun scaffolds.

Samples	Conductivity (S/Cm)
PPC/NMP/9%	9.09 × 10^–6^
PPC/NMP/15%	7.12 × 10^–7^
PPC/NMP/23%	3.7 × 10^–8^
PPC/DMSO/9%	6.24 × 10^–6^
PPC/DMSO/15%	1.46 × 10^–7^
PPC/DMSO/23%	1.06 × 10^–8^
PPC/HFIP/9%	3.7 × 10^–5^
PPC/HFIP/15%	4.9 × 10^–6^
PPC/HFIP/23%	7.03 × 10^–8^

**FIGURE 4:**
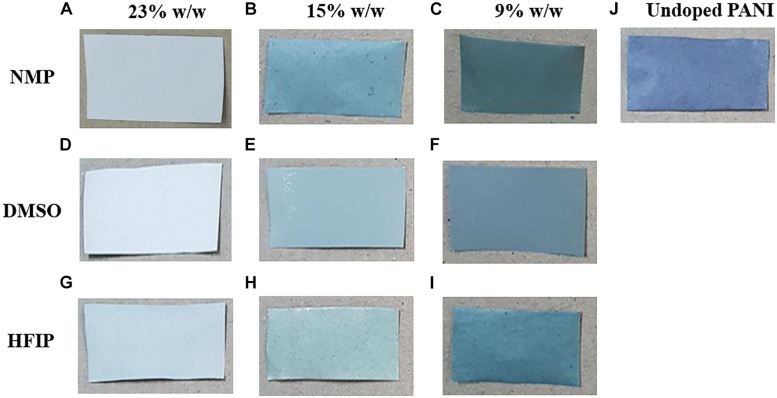
Digital photograph showing the color of PPC nanofibers changing from white to light green and dark green by decreasing the amount of PES in the nanofibers. Nanofibers prepared with NMP **(A–C)**, DMSO **(D–F)**, and HFIP **(G–I)**. PES/Undoped-PANI nanofibers **(J)**.

A simple and easy way to check the percentage of PANI in the scaffold and its conductivity is to compare the colors of the scaffolds. PANI is green in its conductive form. Thus, the darker green scaffold contains more PANI with higher conductivity (Merlini et al., 2016). The different colors of the nanofibers are shown in Figure 4. It can be seen that at the 23% w/w concentration of PES, the color of nanofibers is approximately white resembling the low percentage of PANI in the scaffolds and non-acceptable conductivity of the scaffolds (Table 1). By decreasing the concentration of PES in the solution (15 and 9% w/w) the color of nanofibers changed to green and the conductivity increased.

### Porosity of the Nanofibers

**FIGURE 5:**
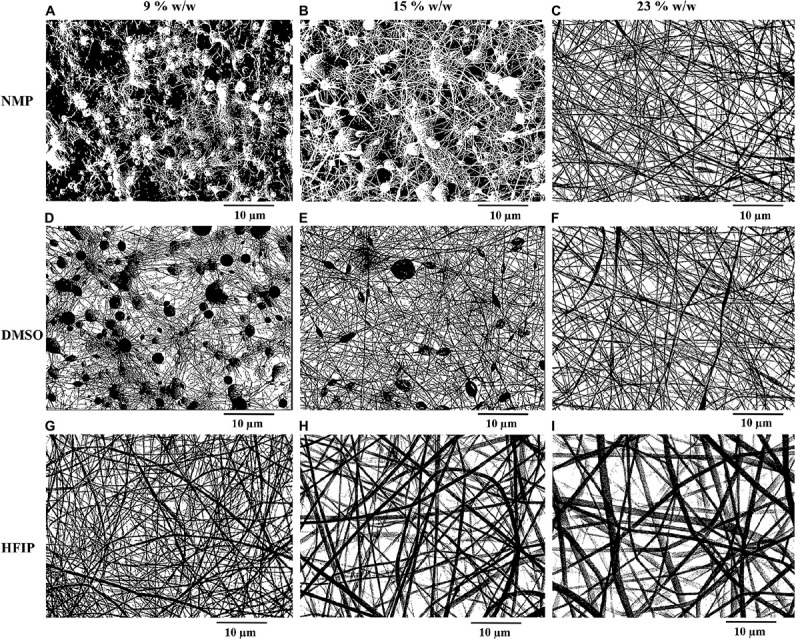
Binary images of electrospun PPC nanofibers prepared with NMP **(A–C)**, DMSO **(D–F)**, and HFIP **(G–I)** in different concentrations of PES [9% w/w **(A,D,G)**, 15% w/w **(B,E,H)**, 23% w/w **(C,F,I)**], (PANI concentration: 0.5% w/w).

Table 2 represents the porosity and average pore area of electrospun nanofibers. Binary images of the nanofibers are also displayed in Figure 5. The porosity was estimated from liquid intrusion, gravimetry, and image processing methods. Different values for porosity were obtained from the three methods.

**TABLE 2:** Porosity and average pore area of electrospun nanofibers.

Samples	Porosity % (Liquid Intrusion)	Porosity % (Gravimetry)	Porosity % (Image)	Average pore Area (μm^2^)
PPC/NMP/9%	–	–	–	–
PPC/NMP/15%	–	–	–	–
PPC/NMP/23%	50.32 ± 4.25	42.61 ± 3.04	54.96	0.2236
PPC/DMSO/9%	10.8 ± 1.98	13.76 ± 32	52.49	0.1875
PPC/DMSO/15%	36.06 ± 3.41	31.4 ± 2.06	52.6	0.179
PPC/DMSO/23%	53.89 ± 2.05	61.13 ± 0.5	56.46	0.2465
PPC/HFIP/9%	89.7 ± 3.2	92.81 ± 1.23	45.33	0.168
PPC/HFIP/15%	83.76 ± 4.31	78.09 ± 3.08	49.37	0.2707
PPC/HFIP/23%	84.92 ± 3.08	83.45 ± 2.9	50.34	0.1643

In the mats prepared with HFIP, it was perceived that as the concentration of PES increased, the porosity and average pore area of the scaffolds decreased. The lower the PES percentage, the thinner nanofibers deposited. Thinner nanofibers offered higher porosity and larger average pore area. In the samples prepared with DMSO, the opposite results were achieved. The porosity increased by increasing the percentage of PES in the mats due to the lower beads existing in the nanofibers. Furthermore, there was a significant difference between the porosity of PPC/DMSO and PPC/HFIP samples (*p* < 0.05). The former had less porosity than the latter due to the fluffy structure and larger pore area of the PPC/HFIP samples.

It was not possible to measure the porosity of the nanofibers prepared with NMP in 9 and 15% w/w of PES concentrations because a large part of the scaffolds was made of polymeric film and there were very small nanofibers. The numbers obtained through image analysis were not realistic for these samples because it considered the polymeric film as pores (Figures 5A,B). The porosity of PPC/NMP/23% sample was significantly lower than PPC/HFIP/9% (*p* < 0.05).

### Cell Morphology on the Nanofibers

**FIGURE 6:**
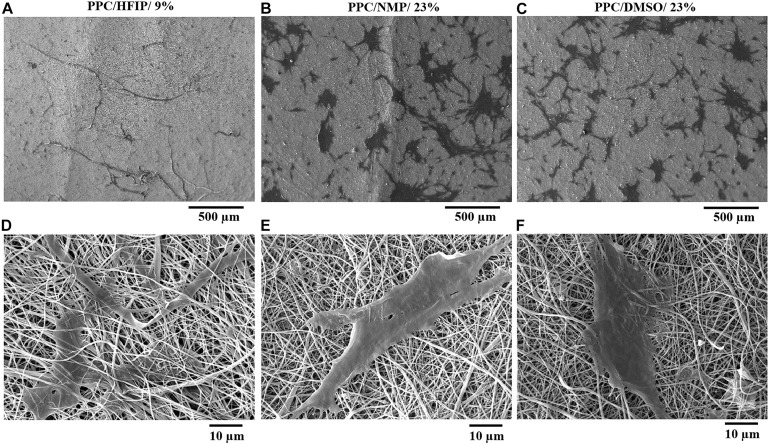
SEM micrographs of 3T3 cells adhesion, morphology, and penetration in PES/PANI nanofibers after 48 h of culture. **(A,D)** PPC/HFIP/9%. **(B,E)** PPC/NMP/23%. **(C,F)** PPC/DMSO/23%.

Cell adhesion to scaffolds and cell infiltration into nanofibers were investigated using SEM images. The SEM micrographs of the cell-scaffold structures after 2 days of cell culture are shown in Figure 6. The appropriate spreading of the 3T3 cells on the nanofibrous scaffolds was observed. The cells appeared to be more spread on PPC/HFIP/9% sample.

As it is shown in Figures 6D,F, the cells were adhered to the surface of the nanofibers while in Figure 6B the cells have passed the superficial nanofibers and infiltrate to a certain depth of the PPC/HFIP/9% scaffold. Comparison of the cell penetration to the three nanofibrous scaffolds was more tangible in Figures 6C–E at 500 μm magnification. Most cells penetrated the nanofibers and the cells were not visible on the surface of the fluffy scaffold in Figure 6A. A lot of cells are visible on the surface of sheet-like mats with densely packed nanofibers indicating low cellular infiltration in those scaffolds.

### MTT Assay

**FIGURE 7:**
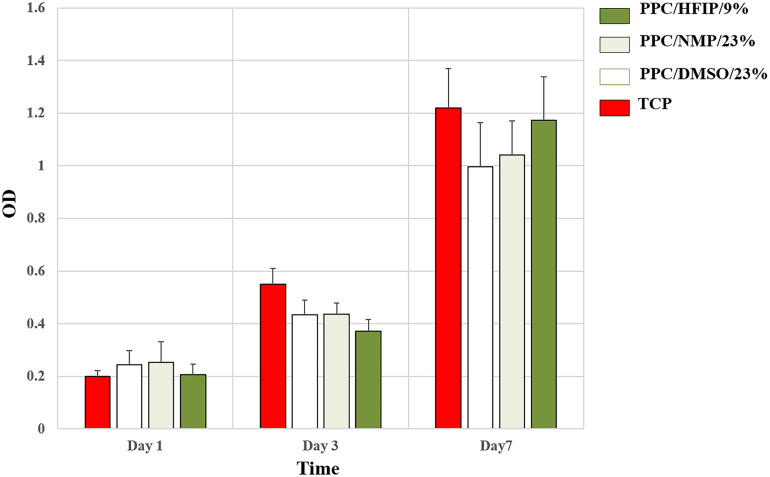
MTT cell proliferation assay of 3T3 cells on scaffolds and TCP during the 7-day culture period. Data are expressed as mean ± SD. The difference between quantities is not significant (*p* > 0.05).

The ability of conductive nanofibers to support cell proliferation was evaluated via MTT assay. As it was shown in Figure 7, all groups supported cell proliferation over 7 days, and the OD of all samples indicating the number of living cells on them was not significantly different. In fact, no scaffold caused cytotoxicity. However, the difference in cell proliferation in the PPC/HFIP/9% sample with the other two conductive samples containing PANI was noteworthy. Unexpectedly, the number of living cells on the 3D sample was less than the ones on the 2D samples until day 3. On the other hand, the viability of cells on the 3D nanofibers was more than 2D nanofibers on day 7.

## Discussion

As it was mentioned earlier, solution properties play the most important role in the electrospinning of a polymer. The solutions with higher viscosity and conductivity, as well as the solvents with the lower surface tension and the boiling point assist the formation of nanofibers without beads (Fong et al., 1999). On the other hand, the electrical properties of the final nanofibers besides the morphology must also be considered for electrospinning of PANI-based nanofibers. The conductivity of the composite PANI-based nanofibers is affected by the amount of carrier polymer, the solubility of PANI in the solvent, and the effect of solvent on doping of PANI (Yanılmaz and Sarac, 2014). The solubility of PANI in NMP and HFIP is more than DMSO. Among the three solvents, only HFIP acts as a secondary dopant for PANI and improves PANI conformation from coil-like to straight chain (Yonehara and Goto, 2020). As a result, HFIP seems to be more suitable than other solvents for dissolving PANI. On the other hand, electrospinning of PES/HFIP solution can be performed at lower concentrations compared to DMSO and NMP. Therefore, the conductivity of PPC/HFIP/9% composite nanofibers was higher due to the less amount of non-conductive carrier polymer.

In present study, the PES concentration should be increased for increasing the solution viscosity. However, increasing the concentration of PES decreases the percentage of PANI in the final solution and results in the reduction of solution conductivity. Furthermore, the solubility of PANI is very low and it is almost impossible to make a PANI solution with a concentration above 0.5% w/v. Therefore, the percentage of carrier polymer should be reduced to result in a solution with a higher percentage of PANI. By decreasing the percentage of PES in PPC/NMP and PPC/DMSO samples, the solutions with lower viscosity are obtained and lead to the formation of beads and particles. Solution viscosity determines the amount of polymer chain molecules entanglement in the solution. The solutions with low viscosity (9 and 15% w/w PES concentration in DMSO and NMP) had low viscoelastic forces during electrospinning, which were not able to match the electrostatic and columbic repulsion forces that stretch the electrospinning jet. This led to jet partially break up. However, electrospinning was also possible at low concentrations by changing the solvent to HFIP. This was attributed to the different properties of the solvents (Table 3).

**TABLE 3:** Properties of NMP, DMSO, and HFIP.

Solvents	Surface Tension (mN⋅m^–^^1^)	Viscosity (mPa⋅s)	Boiling Point (°C)	Dielectric Constant (F/m)
DMSO	43.54	1.99	189	46.7
NMP	41	1.8	202	33
HFIP	16.1	1.65	58.2	16.7

Polyether sulfone (Mw∼58,000) can be electrospun at 25% w/w in solvents such as DMSO (Mohammadi Amirabad et al., 2017) and NMP (Yoon et al., 2009). But electrospinning of PES in HFIP is possible at concentrations even lower than 9% w/w (Zonca et al., 2014). The surface tension of HFIP is much lower than DMSO and NMP. Moreover, the boiling point of HPIF is 58.2°C while for DMSO and NMP it is 189°C and 202°C, respectively. That is why HFIP as a volatile solvent with low surface tension is more suitable for producing PPC nanofibers in low concentrations. It has been shown that solvents with lower surface tension have the highest ability for electrospinning of nanofibers (Pattamaprom et al., 2006). In solvents with high surface tension, the high numbers of free solvent molecules in the solution come together into a spherical shape causing the formation of beads and particles. To solve this problem, the concentration of the solution should be increased., An increase in polymer chain entanglement occurs when solution concentration is increased, and the solvent molecules can be distributed over the entangled polymer molecules leading to the formation of smooth nanofibers (Tungprapa et al., 2007; Casasola et al., 2014). Therefore, the solution concentration at which smooth nanofibers can be achieved for HFIP with lower surface tension was higher for DMSO and NMP, which had higher surface tensions. On the other hand, volatile solvents are the preferred choices as they facilitate dehydration of the nanofibers during trajectory from the needle to the collector surface owing to their lower boiling point and hence rapid evaporation rate. Evaporation during the electrospinning process causes an increase in jet viscosity and inhibits bead formation. So, electrospinning of the low concentration solutions would be possible with volatile solvents (Qian et al., 2010; Golecki et al., 2014).

Any factor contributing to the electrospinnability of a polymeric solution also plays a role in increasing the diameter of nanofibers. Since those factors prevent the electrospinning jet from rupturing, they inhibit nanofibers from stretching and reducing their diameter. As a result, HFIP with lower surface tension and boiling point led to the production of nanofibers with larger diameters. Besides, solutions obtained from the solvent with a lower dielectric constant are expected to have lower conductivity. This contributes to a lower repulsive coulombic force within the jet segments, resulting in lower stretching of the charged jet during its flight between the needle and the collector. On the other hand, the presence of PANI-CSA in the PES solution is like adding salt to the electrospinning solutions, which increases the ionic conductivity and dielectric constant of the solutions (Prabhakaran et al., 2011). Although HFIP has a low dielectric constant, the presence of PANI-CSA should provide the necessary conductivity for electrospinning and stretching of the nanofibers.

High SD from the mean diameter may occur for two reasons including low or high viscosity of the polymeric solution. In the solutions prepared with DMSO and NMP, even in 23% w/w PES concentration, it was impossible to produce uniform nanofibers and some cylindrical beads in the nanofibers were formed. This caused high SD in the diameter of nanofibers of PPC/DMSO/23% and PPC/NMP/23% (Even with the change of other electrospinning parameters, the preparation of bead-free nanofibers was not possible). Besides, 23% w/w PES concentration in PPC/HFIP/23% is too high and resulted in the formation of multi-branched jets with different diameters during electrospinning of the solution (Deitzel et al., 2001; Gu et al., 2014b).

Bead-free nanofibers with suitable diameter distribution, and a 3D structure with appropriate porosity are required to prepare nanofibers scaffolds simulating natural ECM. A major problem in electrospinning is the tendency of nanofibers to accumulate densely, resulting in a 2D mat with poor porosity and small pore size. The packing density of the nanofibrous mats could be modulated through changing electrospinning parameters such as changing the electric charge of the solution (Sun et al., 2012), redirection and deceleration of electrospinning jets (Miyamoto et al., 2009), and changing the collector design (Blakeney et al., 2011). Post-electrospinning modification techniques such as ultrasonication (Aghajanpoor et al., 2017) and gas-foaming (Jiang et al., 2015) modulate the packing density of mats as well. Solvent evaporation rate has an important effect on the morphology of nanofibers. The boiling point of HFIP is significantly lower than DMSO and NMP. So, the formation of fluffy nanofibers can be attributed to the faster solvent evaporation. The solvent evaporation rate can be controlled by changing solvent volatility and environment temperature. In our previous study, it was shown that ambient temperature can affect the evaporation rate of the solvent and may modify the creation of a 3D nanofibrous structure (Shabani et al., 2012). In present study the effect of solvent volatility on the formation of fluffy 3D nanofibers is presented. Moreover, between the three scaffolds prepared with HFIP, only PPC/HFIP/9% sample is in 3D form due to the higher percentage of PANI in this solution. Although all the solutions containing doped-PANI are conductive, in a critical concentration the electrical conductivity is greatly enhanced. More conductive solutions transport more electrical charges to the collector. These extra charges reduce the attractive forces between new nanofibers and the collecting region. Furthermore, the electrical charges of lots of nanofibers become similar to the collecting region charge. Then, the attractive forces between the collected nanofibers and needle will be extremely increased. Increasing the attractive forces between nanofibers and needle as well as decreasing the forces between nanofibers and collector result in decreasing nanofibers stacking density. Furthermore, like-charge repulsion between neighboring nanofibers is another possible reason for the nanofibers fluffing. Higher electrostatic repulsions among nanofibers during drying and deposition contribute to obtain a more open nanofibrous scaffold (Khorshidi and Karkhaneh, 2016).

The porosity and pore sizes in the electrospun scaffolds are mainly dependent on the diameters of nanofibers and their packing density (Soliman et al., 2011). The effect of diameter and compaction on porosity are shown in the liquid intrusion and gravimetry methods. However, porosity is more based on the size of the surface nanofibers in the image analysis method, and the compaction effect on it is not measurable. Results showed the porosity of the PPC/HFIP/9% was the highest which could be related to the 3D structure and lower packing density of the nanofibers prepared with HFIP.

Cell morphology is the first cellular behavior that changes according to the environment in which the cells are located and underlies other cellular behaviors. As a result, an easy way to determine the right conditions for the cells is to evaluate their morphology. Cell attachment, penetration, and interaction with the surface of a scaffold can be analyzed according to the cell morphology on the scaffold. In general, nano features improve cell adhesion because the extracellular matrix is composed of nanofibrillar components (Chen et al., 2007). On the other hand, cells have good interaction with conductive substrates due to increased adsorption of positively charged matrix proteins onto the negatively charged surface of conductive polymers (Gu et al., 2014a). Moreover, the adhesive molecules in the membrane of the cells may attach better to the hydrophilic surfaces and may be more stable than hydrophobic ones because of their special molecular structure. According to Figure 6, the cells had the highest elongation and penetration in the PPC/HFIP/9% nanofibers. PES-based substrates are hydrophobic and the presence of PANI in the scaffolds increases their hydrophilicity (Razali et al., 2014). As a result, the higher hydrophilicity along with the higher conductivity gave rise to a better environment for cellular attachment in PPC/HFIP/9% nanofibers.

Despite all the advantages of electrospun scaffolds, these nanofibers have a major limitation. The porosity of electrospun nanofibers is not suitable for cell infiltration into the inner areas of the scaffold (Zhong et al., 2012). There are many ways to overcome this limitation. Chemical (collagen-grafting) and physical (drying of nanofibers to make 3D scaffolds) properties of PES and PLLA nanofibers may be manipulated to enhance cellular infiltration (Shabani et al., 2009, 2012). Similarly in present study, changing the structure of nanofibers from 2D to 3D tried to improve cell penetration into nanofibers. The infiltration was enhanced even though the surface pores were smaller than the size of cells. This shows the smart migration of cells through the loosely packed fluffy nanofibrous structure. At low nanofiber packing density, the porosity of scaffolds is high to allow cells to migrate and proliferate inside the scaffold and to form thick layer. However, when initial nanofiber packing density is high, cells can only migrate and proliferate horizontally and not vertically. Qualitatively, it was obvious that scaffold fabricated with NMP and DMSO had much more dense structure compared to the one fabricated with HFIP. Despite this, as mentioned in previous sections, there was no significant difference between the average pore area and diameter of the nanofibrous scaffolds. According to other studies, reducing the diameter of nanofibers decreases their porosity and cell penetration becomes difficult. But if the nanofibers do not have a compact structure, the cells can smartly push them aside and penetrate into the scaffold. This is necessary for in-growth while the cells are about 10 times larger than pores.

Cell viability on a scaffold depends on the interaction between cells and the structural features and the physicochemical composition of the scaffold. Although, the number of living cells on conductive PPC scaffolds was not significantly different, the results of cell viability in day 3 and 7 draw attention to the cytotoxicity of PANI and the three-dimensionality of the PPC/HFIP/9% scaffold, respectively. Many researchers claimed that PANI shows time- or dose-dependent cytotoxicity (Humpolicek et al., 2012; Kucekova et al., 2014). Li et al. (2014) studied the cytotoxicity of PANI nanomaterial with various concentrations of PANI and different periods ranging from 24 to 72 h on rat celiac macrophages. They reported that the hazardous potential of PANI on macrophages is time- and dose-dependent and high doses of PANI (10 μg/ml or above) can induce cell death (Li et al., 2014). Bagheri et al. (2020) fabricated electrospun scaffold based on chitosan/aniline oligomer/polyvinyl alcohol and represented good cell attachment and proliferation on the surface of the conductive nanofibers. They claimed that this phenomenon was attributed to the enhancement of intercellular activity because of the electroactivity feature of the aniline oligomer. On the other hand, they showed that cell viability on the conductive scaffold drastically decreased with increasing the aniline oligomer content (Bagheri et al., 2020). Various approaches have been used to reduce the cytotoxicity of PANI (Ghasemi-Mobarakeh et al., 2009; Borah et al., 2015; Tan et al., 2018; Zarrintaj et al., 2018). It was demonstrated that the biochemical properties of PANI largely depend on the dopant used for it. The cytotoxicity of PANI-based nanofibers was significantly reduced by replacing CSA with taurine as a bioactive dopant (Daraeinejad and Shabani, 2021). 3D scaffold had higher percentage of PANI than 2D scaffolds. Despite the cytotoxicity of PANI in higher doses, the viability of cells was more on the 3D nanofibers on day 7. This difference can be attributed to the infiltration of cells into the 3D nanofibers and their proliferation in the porosity of the scaffold. This interesting result showed the positive effect of porosity and 3D structure of scaffold on cell proliferation. Rampichová et al. (2013) compared the proliferation of mesenchymal stem cells on 3D and 2D nanofibrous scaffolds. They observed better proliferation and viability of the cells on the 3D nanofibers. They described this result by contact inhibition of cells, which naturally occurs earlier on 2D surfaces than on 3D scaffolds. So, 3D nanofibers offer more space for cell proliferation, especially for long-term cell culture (Rampichová et al., 2013).

Consequently, it can be concluded that the 3D structure of nanofibers can support proliferation and viability of cells and is essential for tissue engineering applications.

## Conclusion

In present work, the effect of solvent on the formation of 3D electrospun PANI-based nanofibers for tissue engineering applications was studied. Three points must be considered to select a suitable solvent for the preparation of PANI/carrier polymer blend nanofibers, including the solubility of the polymers (PANI and carrier polymer) in the solvent, the effect of solvent on the conductivity of PANI, and the properties of the solvent such as boiling point and surface tension. It was shown that among the three solvents used in this study (DMSO, NMP, and HFIP), HFIP was the best option for the fabrication of PANI/PES nanofibers. PES could be electrospun at a concentration of 9% w/w in HFIP, while the optimum percentage of PES in DMSO and NMP was above 23% w/w to produce uniform nanofibers. The solvent was selected in such a way that electrospinning was possible with the minimum carrier polymer and maximum PANI concentration because a lower amount of carrier polymer (non-conductive component) made nanofibers with higher conductivity. Furthermore, it was represented that the higher conductivity of the solution and the higher volatility of the solvent led to the formation of 3D fluffy nanofibers. The resulting 3D conductive nanofibers showed higher cell infiltration than the 2D mats on day 7th. This scaffold had potential application for tissue engineering of electroactive tissues such as nerve, muscle, heart, and bone.

## Data Availability Statement

The original contributions presented in the study are included in the article/supplementary material, further inquiries can be directed to the corresponding author.

## Author Contributions

IS: supervision, project administration, funding acquisition, writing – review and editing, and visualization. ZD: conceptualization, methodology, software, validation, formal analysis, investigation, resources, data curation, writing – original draft, and visualization. Both authors contributed to the article and approved the submitted version.

## Conflict of Interest

The authors declare that the research was conducted in the absence of any commercial or financial relationships that could be construed as a potential conflict of interest.
